# Seasonal home ranges and habitat selection of three elk (*Cervus elaphus*) herds in North Dakota

**DOI:** 10.1371/journal.pone.0211650

**Published:** 2019-02-04

**Authors:** Jacqueline M. Amor, Robert Newman, William F. Jensen, Bradley C. Rundquist, W. David Walter, Jason R. Boulanger

**Affiliations:** 1 Department of Geography, University of North Dakota, Grand Forks, North Dakota, United States of America; 2 Department of Biology, University of North Dakota, Grand Forks, North Dakota, United States of America; 3 North Dakota Game and Fish Department, Bismarck, North Dakota, United States of America; 4 United States Geological Survey, Pennsylvania Cooperative Fish and Wildlife Research Unit, The Pennsylvania State University, University Park, Pennsylvania, United States of America; Texas State University, UNITED STATES

## Abstract

Changes in land use have resulted in range shifts of many wildlife species, including those entering novel environments, resulting in the critical need to understand their spatial ecology to inform ecosystem effects and management decisions. Dispersing elk (*Cervus elaphus*) were colonizing areas of suitable habitat in the Northern Great Plains, USA, resulting in crop depredation complaints in these areas. Although state resource managers had little information on these elk herds, limited evidence suggested temporal movements into Canada. We collected and analyzed essential information on home range and habitat selection for 3 elk herds residing in North Dakota. We captured 5 adult female elk in each study area, affixed global positioning system collars, and monitored them for 1 year (2016–2017). We estimated diel period, seasonal, and hunting season home ranges using Brownian Bridge Movement Models for each individual. We analyzed habitat selection using multinomial logit models to test for differences in use of land classes, and for departures from proportionate use based on random sampling; our predictor variables included individual elk, diel period, and season. Home ranges differed between the 3 herds, seasons, and diel period; gun and winter season home ranges were both larger than in summer, as was night when compared with day. Female elk generally restricted themselves to cover during the day and entered open areas at night and during winter months. Our results also suggest that elk in our study areas tended to seek more cover, and in the case of our Turtle Mountain study area, some cross into Canada during gun season. Our study provides a better understanding of the spatial ecology of elk in the Northern Great Plains while highlighting the need for enhanced international cooperative management efforts.

## Introduction

Elk (*Cervus elephus*) were extirpated from most of their range [1][2][3], including North Dakota, near the end of the 19^th^ century [4]. Since then, elk have been reintroduced into historical locations and have expanded their range [5]. Their reintroduction and dispersal from historical range has led to recolonization of areas with suitable habitat [6], and today, elk are again among the most widely distributed member of Cervidae in North America [7]. In some areas, elk are overabundant, which can lead to human-wildlife conflicts such as property damage, crop depredation, car collisions, and disease transmission, which may result in lower landowner tolerance, especially from growers experiencing crop depredation [8][9][10][11].

Elk conservation and management success is particularly dependent on knowledge of their spatial ecology [12]. For example, by studying movements of individual elk, researchers gain insight into population distributions, resource use, dispersal strategies, social interactions, and general patterns of space use [13]. In addition, habitat resource selection functions (RSFs), defined as any function proportional to the probability of use of a resource unit or unit area by an animal [14], can provide insights on resource-use patterns that influence survival and fitness in various habitats [15]. Home range size may be affected by body size, sex, age, and landscape heterogeneity [16]. Moreover, elk movements and habitat use may vary by geographic location, available cover, season, and diel period [17][18]. For example, elk may have smaller home ranges in areas with a high percent of forest cover, because forest cover is an important resource for reducing predation risk [19][20]. Researchers may assess seasonal time periods because of changing habitat conditions, biology (e.g., calving), or anthropogenic forces such as hunting. For example, reduced habitat quality due to snow and cold temperatures may force elk to travel farther to seek adequate forage and cover [21], while calving season may reduce travel for female elk [22]. Hunting may affect elk distributions, but the timing and degree of changes reported in the literature are not consistent across populations [23]. Others have reported that elk may avoid hunters by moving into areas that offer more protection [24] or increase distance traveled by individual elk [25] during hunting seasons. Inclusion of shorter sampling periods such as diel categories that reflect animal foraging activities, although less common, may improve overall RSFs [18]. Although elk are among the most studied member of Cervidae, supportive data for elk ecology and management has often focused on forested environments in more montane habitats [26][7][27][28]. Less is known about their spatial ecology in prairie regions, with the few studies on resource selection of elk in North Dakota conducted in the western badlands region of the state [29][30][31].

Although elk first reappeared in northeastern North Dakota in the 1970s and have since established herds in other areas of the state [4], in recent years, North Dakota Game and Fish Department (NDGFD) had been receiving increased sighting reports and nuisance complaints within the Turtle Mountain (Bottineau and Rolette counties) and Pembina Hills (Pembina and Cavalier counties) regions of North Dakota that border Canada (W. F. Jensen, NDGFD, personal communication). In addition, sighting reports had been increasing in the Porcupine Hills region (Sioux County) in the southcentral part of the state where elk once were rarely or infrequently observed. The northern elk populations presumably dispersed from established herds in Manitoba, Canada as early as the 1970s and 1980s [4], but the source for the newly established population in the Porcupine Hills region was unclear. The most probable source for elk immigrating into the Porcupine Hills are from established populations in the Badlands region of North Dakota about 200 km to the west. Other potential sources are herds in the Black Hills of South Dakota about 300 km to the southwest, the aforementioned Turtle Mountain and Pembina Hills herds 310 km and 370 km to the north, respectively, or escaped game farm animals from an unknown location (W. F. Jensen, NDGFD, personal communication). Aerial elk counts in Pembina Hills yielded 128 individuals in 2014 [32]. Although aerial counts for Porcupine Hills and Turtle Mountain were unavailable prior to this study, later reports yielded counts of up to 110 elk in Porcupine Hills [33]. Aerial counts in the Turtle Mountain area yielded approximately 62 elk on the U.S. side of the border [33] and subsequently 190 elk when cross-border counts were conducted [34]. These surveys, and incidental reports of small groups of elk outside the home ranges of collared elk reported elsewhere [33], suggested that there were multiple herds of elk in the Turtle Mountain area. As large herd animals, even modest-sized elk herds can cause localized depredation problems [9], particularly when they cross political jurisdictions lines such as international and reservation borders.

We investigated spatial ecology of elk herds in 3 study areas in the Northern Great Plains that varied in landscape composition to expand our understanding of elk ecology in this lesser known portion of their range. Specifically, we 1) estimated female elk seasonal, annual, and diel period home range sizes and differences between these attributes by study area, and 2) analyzed habitat selection at the landscape level in each study area. We hypothesized differences in home range by herd, season, and diel period. Specifically, we predicted larger home ranges from elk residing in more open habitat within the Porcupine Hills study area. We also predicted differences in home range sizes by season and diel period, with those during winter months, hunting season, and nighttime being largest, respectively. Predominant land class types varied among our 3 study areas; therefore, we predicted differences in use of land classes, and for departures from proportionate use as a function of diel period, and season. For example, we predicted that female elk would seek more cover such as forested areas during day and select for more open areas at night during foraging hours. We also predicted that elk would prefer areas of cover in winter.

## Materials and methods

### Study area

**Fig 1:**
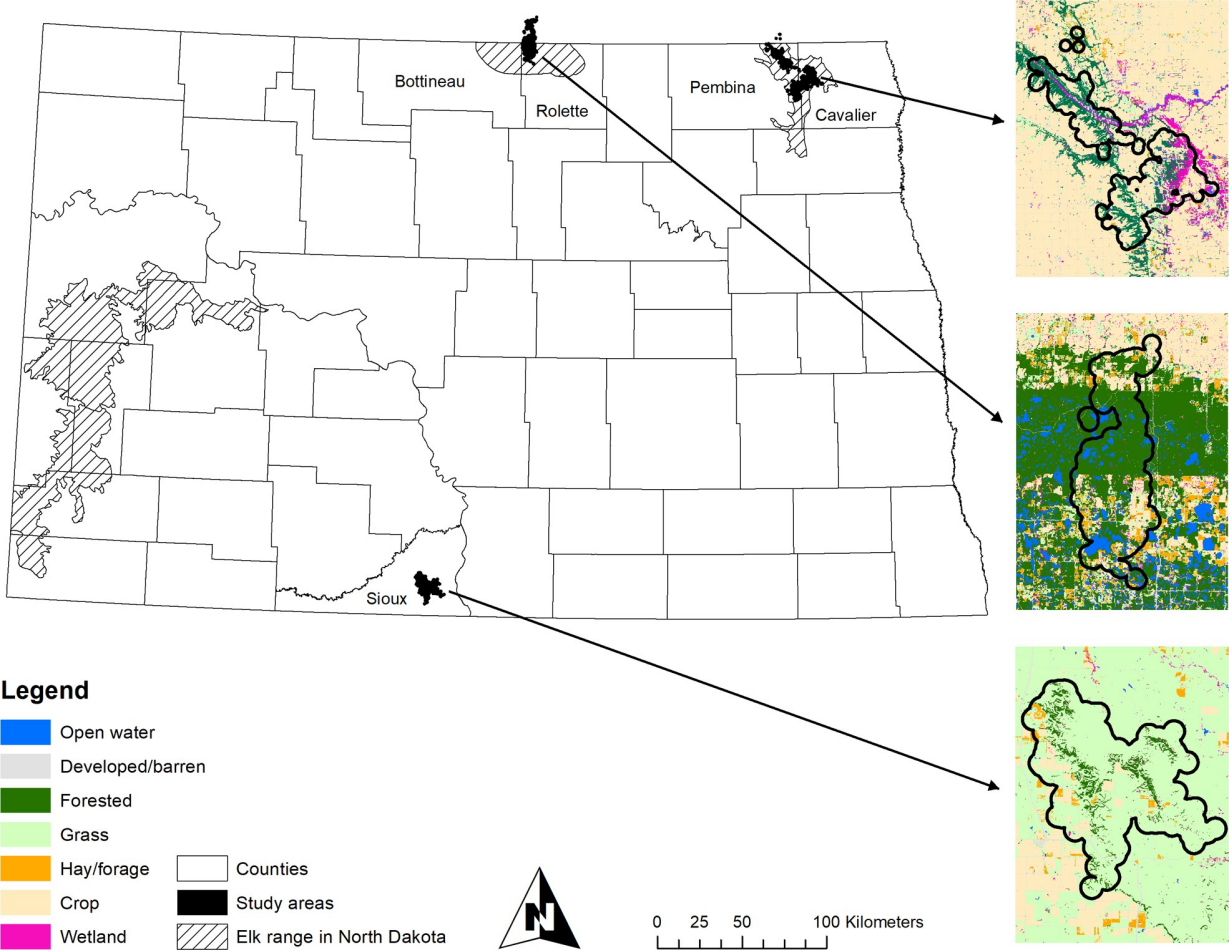
Study area map depicting aggregate elk home ranges with 1 km buffer, available land cover types within buffers, and known elk range in North Dakota prior to this study. Study areas included Turtle Mountain (Bottineau and Rolette counties), Pembina Hills (Pembina and Cavalier counties), and Porcupine Hills (Sioux County) regions, North Dakota, USA, 2016–2017.

We focused efforts in 3 regions: Turtle Mountain region (-100.118613, 49.009258) in the northcentral portion of the state (Bottineau and Rolette counties), Pembina Hills region (-97.978842, 48.843417) in the northeastern portion of the state (Cavalier and Pembina counties), and Porcupine Hills region (-100.838175, 46.095123) in the southcentral portion of the state (Sioux County; Fig 1). Our study areas generally had a continental climate marked by hot summers and harsh, cold winters, with an average annual precipitation of 42.7 cm [2][35]. We compare proportions of available land class types among study areas in supporting information (S1–S3 Figs).

Turtle Mountain, straddles the U.S. and Canadian border, and rises 200–275 m above the surrounding prairie, allowing oak-aspen forests to thrive in these areas [2]. Common tree species include aspen (*Populus tremuloides*), paper birch (*Betula papyrifera*), box elder (*Acer negundo*), and bur oak (*Quercus macrocarpa*) [36][2]. Turtle Mountain is predominately forested, but includes a mosaic of forest, small lakes and wetlands, and agriculture fields, with a somewhat greater proportion of cropland on the U.S. side of the border [2]. The Canadian portion of Turtle Mountain is managed as a public recreation area (Turtle Mountain Provincial Park) primarily comprised of oak-aspen forest. Pembina Hills are similar in types of vegetation to Turtle Mountain, but differ substantially in representation, with crop and agriculture fields more prevalent. While the elevation range is 266 m in Pembina Hills [37], characteristic of this area are coulees (deep ravines) and rivers; downcutting of these rivers also differentiates this terrain from Turtle Mountain. Porcupine Hills is a drier region of highly eroded areas, steep escarpments and buttes, rising 120 m above surrounding plains. Specifically, this landscape consists of woody draws of ash and bur oak forests, eroded draws, and surrounding mixed and shortgrass prairie, including blue grama (*Bouteloua gracilis*), western wheatgrass (*Pascopyrum smithii*), prairie Junegrass (*Koeleria macrantha*), needle-and-thread (*Hesperostipa comate*), needleleaf sedge (*Carex duriuscula*), yarrow (*Achillea millefolium*), gumweed (*Grindelia squarrosa*), silver sage (*Salvia argentea*), and prickly pear cactus (*Opuntia polyacantha*) [2][35]. Both plant community composition and overall makeup of the landscape distinguish Porcupine Hills from the other study areas.

Elk in North Dakota are managed by NDGFD via hunting, and most hunting activities occur on private lands given 93% of land within the state is held under private ownership (NDGFD, https://gf.nd.gov/private-lands, accessed 20 December 2018). During fall 2016, Turtle Mountain and Pembina Hills study areas fell within a single elk management unit where elk-archery hunting season ran from 2 to 25 September, and elk-gun hunting season ran from 7 October to 31 December; 101 available elk permits were distributed to hunters via lottery. Porcupine Hills was closed to elk hunting during the fall of 2016. Statewide archery and gun seasons for deer, including the Porcupine Hills, ran from 2 September 2016 to 8 January 2017 and 4 to 20 November, respectively. In Canada, limited hunting was available within Turtle Mountain Provincial Park and the surrounding area via lottery during an archery (29 August to 18 September) and rifle season (19 to 24 December); 45 available elk permits were distributed to hunters in 2016. Archery and gun seasons for deer in Turtle Mountain Provincial Park ran from 5 September 2016 to 13 November 2016 and 14 to 27 November, respectively.

### Capture

In February 2016, we captured 15 adult female elk via helicopter (Native Range Capture Service, Elko, Nevada, USA) and net-gun [38] on private and state lands. We generally focused capture and collaring efforts in areas central to known elk range or reported nuisance complaints or sightings. Upon capture, elk were restrained, blindfolded, and processed without anesthesia to reduce handling time. We fitted captured elk with livestock ear tags (Premier1 Supplies, Washington, IA, USA) and global positioning system (GPS) tracking collars capable of <3 m location accuracy (Iridium Model #G2110E, Advanced Telemetry Systems, Minnesota, USA). We programmed collars to record a GPS location fix every 4 hours, providing 6 locations per day and estimated battery life through May 2018. All aspects of elk capture and handling protocols followed the general guidelines of the American Society of Mammalogists [39] and complied with UND’s animal care standards (Animal Welfare Assurance Number A3917-01 and Institutional Animal Care and Use Committee Protocol Number 1602–1). This research was approved by NDGFD (Study No. C-XI).

### Home range

We used Brownian Bridge Movement Models (BBMM) [40] to estimate home ranges for each female elk and time period of interest. BBMM accounts for proportional intensity of area use and non-independence of observations over short time periods, assuming that movements are random during the interval between fixes [41][42]. Because we wished to test whether the extent of elk space use varied by time of day and year, we estimated home ranges for each female elk separately for each time period of interest, including diel period (daytime and nighttime) and during 5 consecutive, but non-overlapping, biologically or management-defined seasons. Diel period was determined based on a sun angle calculation of sunset and sunrise as a function of latitude and longitude, and time of year. We included the following seasons for analysis: calving (May 1–June 30), summer (July 1–August 31), archery elk hunting season (September 1–September 30), gun elk hunting season (October 1–December 31), and winter (January 1–April 30). We included April in the winter season to accommodate calving season. We estimated BBMM using both 50% and 95% isopleth contours in the R package adehabitatHR R version 3.3.2, https://www.r-project.org/, accessed 10 January 2017) [43]. We used scripts from the Manual of Applied Spatial Ecology [44] for all home range estimates.

To test for differences in home range size (response) among herds, and effects of time of day and season, we used a general linear mixed effects model [45] with individual female elk, nested in herds, treated as a random effect to account for repeated measures on individuals. Our primary focus for this component of space use, however, was discerning differences among herds and effects of specific times and seasons, and not on idiosyncratic behavior of individual elk. All other factors were considered fixed effects, for which estimates of effects would be generated. We considered herd, season, and time of day as categorical factors, requiring the designation of a baseline against which to compare other levels. We used summer as the baseline for comparing seasonal effects, daytime as the baseline for diel period, and Pembina Hills herd as the baseline for herd differences, only because the Pembina Hills herd has been established for the longest period of time (ca. 1975). We analyzed log_10_ transformed home range estimates to meet the assumptions of residual normality. We used R and package nlme (R version 3.3.2, https://www.r-project.org/, accessed 10 May 2017) to estimate models with main effects and two-way interactions to test for differences among factors that depended on other factor levels. We did not consider our analysis as a model selection problem because our goal was to test the effects of all of the included factors and not simply to produce a single or set of models that yielded the optimal prediction of home range size.

### Habitat classification

We used 2016 USDA National Agriculture Statistics Service’s (NASS) Cropland Data Layer (CDL) [46][47] to acquire habitat data within the U.S. We derived habitat data for Manitoba, Canada from the 2016 Agriculture and Agri-Food Canada (AAFC) Annual Crop Inventory (ACI) [48]. We reconciled land classes that differed between U.S. and Canada data sets given that CDL grouped grass and pasture, with hay as a separate class, while ACI grouped pasture and forage, with grass as a separate class. Therefore, we recoded data to the following classes used in initial analyses: open water, developed/barren, forested, shrub, grass, hay/forage, crop, and wetland. However, we subsequently removed shrub from the analysis due to its infrequent occurrence across all study areas (1.4% overall). We also conducted a habitat accuracy assessment by visually checking 500 random points against reference data in the form of 2016 true color National Agriculture Imagery Program (NAIP) and 2015 DigitalGlobe imagery available through ESRI World Imagery for U.S. and Canadian territory, respectively. Both NAIP and DigitalGlobe offer comparable resolution: 2016 North Dakota NAIP is 60 cm and 2015 ESRI DigitalGlobe is 50 cm. We performed analysis, which yields a $\hat{K}$ statistic quantifying actual agreement between a land-cover classification and reference data to expected agreement between the data sets by chance [49]. $\hat{K}$ greater than 0.8 indicate highly accurate, non-random, classifications and those between 0.4 and 0.8 are moderately accurate [50]. $\hat{K}$ values and overall accuracy were 0.734 (84.9%) for Porcupine Hills, 0.832 (89.0%) for Turtle Mountain, and 0.805 (91.2%) for Pembina Hills. Accuracies among forested (31–75%), wetland (33–85%), hay (33–68%), and developed (36–95%) land classes varied more than others. Accuracies for crop (83–98%), grass and pasture (64–98%), and open water (89–100%) were all relatively high among the three study areas.

### Habitat selection

Our analysis of habitat selection centered on the landscape composition where female elk were spending time versus where they were not within each of the 3 regions of interest (Fig 1). Accordingly, we focused on inference at the landscape level (2^nd^-order selection) [51] for the entire set of individual elk to gain an understanding of broader scale, herd level behavior over the course of a year. The 3 sites were analyzed separately because landscape composition differed dramatically between them. We limited our analysis of habitat selection to the frequency of land classes within a 1 km buffer around the area defined by the entire set of GPS locations for all elk in an area for each of the 3 sites, irrespective of time of day, season, or individual elk. However, we compared the scaling of land cover by sampling random points inside buffers of 1 km, 2 km, 4 km, and 8 km. The purpose of this comparison was to judge the potential for inference of habitat selection to be qualitatively altered at different scales, perhaps either becoming less apparent or reversed in direction. If landscape composition did not change much across scales, then clearly neither would the results of the resource selection analysis. However, if landscape composition changed such that a land class that appeared overrepresented at elk locations (forest) declined in overall representation on the landscape as the scale increased, as it appears to do as reported in supplemental information (S1–S3 Figs), then a finding of non-random association at the 1 km scale would be even more strongly supported at the larger extent. We conducted Poisson regression of point counts as a function of buffer extent and land class, and these results indicated that landscape composition in all areas shifts away from forest towards greater proportion of non-forest classes as buffer size expands away from elk locations. The specific changes varied among the 3 areas and in different ways for different land classes, but always in a manner that would accentuate rather than diminish the visually apparent bias towards forest use by elk.

To achieve our objective of determining how female elk use habitat in relation to landscape-level availability, we drew random points equal to the combined number of GPS fixes taken for all elk from each area within 1 km buffer surrounding observed locations. We analyzed habitat selection within these boundaries using multinomial logit models [52][53] to test for differences in use of land classes, and for departures from proportionate use based on random sampling. Using R and multinom in package nnet (Version 3.3.2, www.r-project.org, accessed 28 March 2018) [54], we developed and compared multi-factor models using a model selection approach [55]. Our response variable was land class associated with each location (elk observation or random point). Predictor variables were all categorical fixed effects and included elk ID (5 categories per herd), diel period (day or night), and season (summer, archery, gun, winter, calving), and any two-way interaction terms. We treated elk ID as a fixed factor because we have only a small sample of elk in each area and areas (herds) were analyzed separately. We used land class at random locations in aggregate as the baseline reference for contrast to each level of each predictor variable. Prior to modeling, we examined a correlation matrix for all covariates to screen for collinearity. We used a Wald statistic to test significance of coefficients (α = 0.05) and estimated strength of model fit by comparing residual deviances to null deviances. To aid in visualization of contributions of main effects to model prediction, we present in supplemental information (S4–S11 Figs) effects plots, or back-transformed probabilities of observations of each land class generated using the R package effects [56].

## Results

### Home range

We collected 36,051 GPS locations from 15 GPS-collared female elk during February 2016–April 2017 and present means for study areas by herd, season, and diel period (Tables 1 and 2). During our study, 2 collared elk were harvested during gun season, precluding winter home range estimates for these animals. The largest annual 95% and 50% isopleth contours for herd home range came from the Porcupine Hills elk herd at $\bar{x}$ = 31.9 km^2^ (95% CI: 27.9–35.9) and $\bar{x}$ = 5.6 km^2^ (95% CI: 4.7–6.5), respectively (Table 1). The largest 95% isopleth contour home range for season was from the Pembina Hills herd during gun season at $\bar{x}$ = 47.5 km^2^ (95% CI: 34.5–60.6; Table 1). The largest 50% isopleth contour home range for season was from the Porcupine Hills herd during gun season at $\bar{x}$ = 8.3 km^2^ (95% CI: 6.5–10.1; Table 1). The largest home range for diel period was from the Pembina Hills herd during the nighttime at $\bar{x}$ = 36.9 km^2^ (95% CI: 26.2–47.5; Table 2). Home range estimates for all Turtle Mountain elk included location fixes in Canada, and this mostly occurred during gun season [33]. Only 1 female elk in the Pembina Hills herd was observed crossing into Canada, and this occurred during calving season.

**Table 1:** Mean 95% and 50% female elk home range size (km^2^) by herd and season (including annual), North Dakota, USA during 2016–2017.

		95% Home Ranges				50% Home Ranges			
				95% CI				95% CI	
Herd	Season	Mean	SE	Lower	Upper	Mean	SE	Lower	Upper
Turtle Mountain	Calving	10.8	0.9	8.7	13.0	2.0	0.3	1.4	2.6
	Summer	8.7	1.0	6.5	11.0	1.7	0.2	1.2	2.3
	Archery	11.6	2.7	5.5	17.7	2.3	0.5	1.1	3.5
	Gun	46.1	6.6	31.2	61.0	7.0	0.9	4.8	9.1
	Winter	14.6	1.6	10.9	18.3	2.2	0.1	1.9	2.4
	Annual	18.5	2.6	13.4	23.7	3.1	0.4	2.3	3.8
Pembina Hills	Calving	23.1	7.9	5.06	41.2	3.6	1.1	1.1	6.0
	Summer	22.9	6.0	9.3	36.6	3.8	0.9	1.8	5.9
	Archery	13.7	2.2	8.8	18.7	2.1	0.4	1.1	3.0
	Gun	47.5	5.8	34.5	60.6	7.9	1.1	5.3	10.5
	Winter	43.7	7.4	26.2	61.3	5.6	1.1	2.9	8.3
	Annual	29.5	3.2	23.1	36.2	4.5	0.5	3.5	5.6
Porcupine Hills	Calving	30.7	4.5	20.5	40.8	6.3	1.3	3.5	9.2
	Summer	31.5	5.0	20.1	42.9	4.7	0.7	3.2	6.2
	Archery	19.5	2.2	14.5	24.4	2.9	0.5	1.9	4.0
	Gun	43.3	5.0	32.0	54.6	8.3	0.8	6.5	10.1
	Winter	34.6	1.2	31.9	37.4	5.8	0.4	4.8	6.7
	Annual	31.9	2.0	27.9	35.9	5.6	0.4	4.7	6.5

**Table 2:** Mean 95% and 50% female elk home range size (km^2^) by herd and diel period, North Dakota, USA during 2016–2017.

		95% Home Ranges				50% Home Ranges			
				95% CI				95% CI	
Herd	Diel	Mean	SE	Lower	Upper	Mean	SE	Lower	Upper
Turtle Mountain	Day	15.8	3.7	8.1	23.4	2.6	0.5	1.6	3.6
	Night	21.3	3.5	14.1	28.5	3.5	0.6	2.3	4.6
Pembina Hills	Day	22.4	3.5	15.3	29.6	3.4	0.5	2.4	4.4
	Night	36.9	5.1	26.2	47.5	5.7	0.8	4.0	7.4
Porcupine Hills	Day	30.2	2.5	25.1	35.4	5.2	0.5	4.2	6.3
	Night	33.6	3.2	27.1	40.1	6.0	0.7	4.6	7.4

We found differences (*P* < 0.05) between 95% home ranges and season, diel period, and interactions between study area and season and diel period (Table 3). When compared to Pembina Hills, Turtle Mountain and Porcupine Hills elk home ranges were smaller and larger, respectively. Gun and winter season home ranges were both larger than summer home ranges, as were night in comparison with day. Two-way interactions that differed from baseline included herd by season, with Turtle Mountain elk expanding home ranges during gun season, and Porcupine Hills elk contracting during gun season. The Porcupine Hills herd also contracted in their area used during winter. We discerned no interactions in the effect of time of day and season.

**Table 3:** Linear mixed effects analysis discerning differences in elk (*Cervus elaphus*) home range sizes using Brownian Bridge Movement Models by season and diel period. Output includes coefficient estimates and standard errors (SE) using log_10_ transformed data on 95% home ranges in Turtle Mountain (Turtle), Pembina Hills, and Porcupine Hills (Porcupine) study areas, North Dakota, USA (data collected from 2016–17).

	Estimate	SE	DF	t-value	*P*-value
(Intercept)	1.092	0.089	112	12.207	0
Turtle Mountain	-0.294	0.118	12	-2.500	0.0279
Porcupine Hills	0.332	0.118	12	2.825	0.0153
Calving	-0.067	0.104	112	-0.643	0.5217
Gun	0.477	0.104	112	4.589	0
Archery	-0.096	0.104	112	-0.921	0.3593
Winter	0.359	0.110	112	3.250	0.0015
Night	0.263	0.087	112	3.015	0.0032
Turtle Mountain*Calving	0.152	0.127	112	1.195	0.2347
Porcupine Hills*Calving	0.035	0.127	112	0.273	0.7857
Turtle Mountain*Gun	0.277	0.127	112	2.182	0.0312
Porcupine Hills*Gun	-0.292	0.127	112	-2.299	0.0234
Turtle Mountain*Archery	0.216	0.127	112	1.699	0.092
Porcupine Hills*Archery	-0.051	0.127	112	-0.404	0.6868
Turtle Mountain*Winter	-0.130	0.136	112	-0.957	0.3406
Porcupine Hills*Winter	-0.268	0.132	112	-2.033	0.0444
Turtle Mountain*Night	-0.051	0.082	112	-0.619	0.5374
Porcupine Hills*Night	-0.216	0.081	112	-2.659	0.009
Calving*Night	0.063	0.104	112	0.608	0.5444
Gun*Night	-0.110	0.104	112	-1.057	0.2926
Archery*Night	-0.073	0.104	112	-0.702	0.4839
Winter*Night	-0.003	0.108	112	-0.028	0.9778

### Habitat selection

We analyzed habitat selection within landscapes defined by 1 km buffers common to all female elk in each study area. We observed single interval movements well in excess of 1 km, with the maximum observed 8.5, 12.8, and 9.0 km in a single 4 hr interval for Turtle Mountain, Pembina Hills, and Porcupine Hills, respectively. Mean over all herds, including intervals with <25 m net displacement, was 585 m ± 791 SD. By study area, mean movements were 572.4 m ± 268 SD, 646.5 m ± 313 SD, and 538.0 m ± 252 SD for Turtle Mountain, Pembina Hills, and Porcupine Hills, respectively. Movements were highly skewed, with nearly 20% of movements exceeding 1 km and 90^th^ percentile distance of 1.6 km. However, the representation of each land class on the landscapes did not vary much across scales until distances > 8 km were included, as reported in supplemental information (S1–S3 Figs).

#### Turtle mountain

Percent cover of most land classes at the landscape level were similar across spatial extents represented by the tested series of buffers, albeit with decreasing cover of forest and complementary increases in crops at the broadest extent (S1 Fig). Forest was the most common land class on the landscape at all scales in Turtle Mountain, including the 1 km buffer used for habitat selection analysis.

There was variation in habitat use that was not accounted for by included factors, but the top multinomial regression model demonstrated that these factors still accounted for a portion of that variation (null deviance = 42,466.60, residual deviance = 37,150.04). In the single, top-ranked model (AIC = 37,150.04, 2^nd^ ranked model: ΔAIC = 29.4; S1 Table), departures from random land use were best explained by elk ID, diel period, season, and interactions between diel period and season and elk ID and season (Table 4). There was generally little variation among individual elk in land use, but the odds of using crop versus forest was lower for 2 individual elk than would be expected at random. Forest was used with higher probability and lower variance in the day than at night. The odds of using crop versus forest were also lower during archery and winter seasons than would be expected at random, but higher in gun and summer seasons. Among interactions, the odds of using crop versus forest were lower during the day in archery and calving seasons than would be expected at random, but higher in gun and summer seasons (Table 4). The odds of using crop versus forest was lower during the night during calving season than would be expected at random, but higher in archery and winter seasons.

**Table 4:** Coefficient estimates ($\hat{\beta }$) and standard errors (SE) for contributions to log-odds of significant explanatory variables (α = 0.05; based on Wald-statistics) of the multinomial logit regression models of habitat use in Turtle Mountain, Pembina Hills, and Porcupine Hills study areas, North Dakota, USA (data collected from 2016–17). The 3 study areas were analyzed separately; land covers are the response categories that exhibited significantly different odds of use for the listed predictor level, relative to availability. Individual elk differed from other predictor variables in that only a subset was present in each herd; other predictors were tested in all study areas.

	Study areas									
	Turtle Mountain		Pembina Hills				Porcupine Hills			
	Crop		Grass		Wetland		Grass		Crop	
	$\hat{\beta }$	SE	$\hat{\beta }$	SE	$\hat{\beta }$	SE	$\hat{\beta }$	SE	$\hat{\beta }$	SE
Elk D036792	-1.65	0.16								
Elk D036793									0.56	0.05
Elk D036796							-0.40	0.03		
Elk D036799							-0.51	0.03		
Elk D036804	-1.48	0.17								
Elk D036805							-0.55	0.04		
Day	-8.05	0.18	-1.71	0.08	-0.68	0.05	-1.57	0.03	-1.66	0.07
Night			0.64	0.04			-0.36	0.03	0.51	0.05
Archery	-4.71	0.09					-0.61	0.06	0.80	0.06
Calving							-0.35	0.03		
Gun	1.55	0.15			0.57	0.04	-0.47	0.03		
Summer	1.39	0.10								
Winter	-4.62	0.04							-1.36	0.12
Day*Archery	-8.92	<0.01	-0.83	0.22						
Night*Archery	4.21	0.09								
Day*Calving	3.67	0.32								
Night*Calving	-3.35	0.21								
Day*Gun	3.05	0.30								
Day*Summer	4.06	0.21								
Night*Winter	5.28	0.04								

#### Pembina Hills

The Pembina Hills study area was characterized by less forest and more crop, wetland, and grass compared to Turtle Mountain (S2 Fig). The top multinomial regression model fit was modest with a null deviance of 54,685.87 and a residual deviance of 47,414.13. In the single, top-ranked model (AIC = 47,774.13, 2^nd^ ranked model: ΔAIC = 30.8; S1 Table.), land use was best explained by diel period, season, and interactions between diel period and season and elk ID and season (Table 4). The odds of using grass and wetland versus forest was lower during the day than would be expected at random; however, odds of grass use were higher at night. The odds of using wetland versus forest was higher in gun season than would be expected at random. The odds of using grass versus forest was also lower during the day in archery season than would be expected at random. We detected no significant differences among individual female elk in this analysis.

#### Porcupine Hills

In comparison to our other study areas, availability of forest in Porcupine Hills was low, with commensurate higher availability of grass (S3 Fig). The top multinomial regression model fit was modest with a null deviance of 41,660.43 and a residual deviance of 33,673.02. In the single, top-ranked model (AIC = 34,093.02, 2^nd^ ranked model: ΔAIC = 9.3; S1 Table.), land use was best explained by elk ID, diel period, and season. However, no interaction terms were significant (Table 4). The odds of using grass versus forest was lower for 3 individual elk than would be expected by chance, and higher for crop use for 1 elk without a calf, but overall individual variation was slight. Despite a lower proportion of forest availability, female elk were found disproportionately more in forest during the day. The odds of using grass and crop versus forest was lower during the day than would be expected at random. At night, the odds of grass use were also lower, but the odds of crop use were higher. The odds of using grass versus forest was also lower during archery, calving, and gun seasons than would be expected at random, but the odds of crop use were higher in archery season and lower during winter season.

## Discussion

Our results provide a novel contribution to the spatial ecology of elk herds in varying landscapes within the Northern Great Plains. We found differences in size of home range based on season, diel period, and herd among female elk. Porcupine Hills is predominantly mixed and shortgrass prairie, possibly forcing elk to travel farther from forest cover to find adequate forage. In contrast, Turtle Mountain is heavily forested and intermixed with cropland, potentially reducing the necessary travel distance from security cover [21]. Vegetation and landscape differences [2] between our study areas may explain differences in size of home range among all 3 elk herds [57][58]. Home range size increased during winter and at night. During winter, reduced habitat quality due to snow and cold temperatures may have forced elk to travel farther to seek adequate forage and cover [21]. We expected our study elk to display larger home ranges at night, given elk are known to rest and forage in cover throughout the day and forage in more open areas at night [59]. This was corroborated by our habitat selection analysis which show that female elk were generally spending more time in forest cover during daylight hours and entered open areas at night. There were no differences in home range between summer and calving and archery seasons and size of home range was consistently smaller during these three seasons when compared to gun and winter seasons. These differences are likely due to behaviors related to calving, calf presence, abundance of high quality forage, and harem formation by males for breeding for calving, summer, and archery seasons, respectively.

We found differences in habitat use by individual elk, time of day, and season. We noted that elk especially preferred forest in winter. It has been hypothesized that elk diets may be related to winter severity [60]. In some studies, it was suggested that elk in winter tend to choose locally open areas to feed [61], but that snow depth may impede these efforts [62][63]. No selection patterns were observed within differing stages of secondary forest succession for an elk study in Idaho [64]. Others suggested that elk select feeding sites in timber stands or dense vegetation to lessen thermoregulatory demands [65][66] or for ambulatory ease in snow cover [62]. We speculate the latter, in part, given that long North Dakota winters offer elk snow, extreme wind, and some of the coldest temperatures in the U.S. [67].

Our results emphasize the importance of how individual elk space use may vary across geographical range due to available cover in different habitat types, elk biology, and response to hunting pressure [57]. We speculate that hunting may explain some differences in home range and habitat selection within our study. For example, female elk home range was greater for all 3 elk herds during the gun season than any other season, and this is likely due to hunter pressure from both elk and deer-gun hunters, which displace elk from their usual habitat to seek alternative cover and forage [23][24]. Specifically, elk may avoid hunters by moving into areas that offer more protection, such as national parks (if available), densely vegetated areas, and private lands, as summarized elsewhere [68]. Although there was no elk hunting season in the Porcupine Hills in 2016, this elk herd still appeared to have increased size of home range like the other two herds during this time of year. In addition to direct elk hunting pressure, elk distributions may vary during concurrent deer-gun hunting seasons due to resource selection and dependence on available resources [23]. During the archery season (September) Turtle Mountain and Porcupine Hills elk appeared to use crops more than expected, unlike Pembina. Effects of hunting during archery season on elk movements vary based on studies [23], and are thus unclear for our study areas, but we further note that the physiological demands for cow elk to build fat reserves [69] in preparation for winter after the nutritional demands of nursing a calf are important during this time of year [70]. Presumably this shift in habitat use may also be driven by foraging requirements on the cow. Ultimately, elk that flee from hunted areas may complicate management efforts [27], which may be complicated further when elk, such as those in Turtle Mountain, move into Canada during North Dakota’s longer gun season, thus requiring international cooperative management efforts.

Differences in habitat used during night differed between study areas, and this variation likely occurred due to influences of forage and cover. For example, Turtle Mountain had the most forest, followed by the Pembina Hills and Porcupine Hills study areas. The Pembina Hills study area was characterized by less forest that is distributed linearly along the Pembina River drainage system, and more crop, wetland, and grass compared to Turtle Mountain. In the Porcupine Hills, availability of forest was fairly low, with commensurate higher availability of grasslands. Differences in habitat between our 3 study areas highlight the difficulty in making comparisons to other studies, but also expands our understanding of elk behavior in a greater range of situations. Although our study areas varied in forest cover, we note that elk are adaptable, and may meet their year-round forage and cover requirements for survival in non-forested areas [7]. Few studies conducted in North Dakota also limit comparisons, and those regarding habitat use were mostly limited to western North Dakota [29][30][31]. Two studies in the North Dakota Badlands demonstrate starkly different observations. Elk within Theodore Roosevelt National Park, where hunting is not permitted, did not use overhead cover in winter, and foraged primarily on upland grass throughout the year [30]. In another study, 40 km to the north, a hunted elk herd spent a majority of their time in forested areas throughout the day and night [31]. In Pembina Hills, however, diets from hunter-killed elk during fall and suggest that corn (*Zea mays*) comprised 60% of elk’s diet [71]. While a higher proportion of crops was available in our Pembina Hills study area, we did not discern a difference in the odds of using this land cover compared to forest relative to its availability.

## Conclusions

This is the first study to report on three distinct elk subpopulations in the Northern Great Plains and the variability exhibited by these subpopulations in relation to home range and seasonal resource selection. Size of home range and habitat selection for each elk herd was influenced by season and diel period regardless of the distinct habitat compositions each subpopulation occupied. Resource selection during elk and deer gun hunting season allows resource managers to recognize how elk react to and are influenced by hunter pressure regardless of habitat composition. Since 2 of these elk populations border Canada, future studies may benefit from exploring the ecology of elk on both sides of the international border. Our study will also allow resource managers to better focus management efforts on areas more likely to be used by elk. For example, elk in this region may be restricted to small pockets of forested habitat common in riparian corridors in the Great Plains. How these fragmented landscapes affect elk viability is unknown but may be problematic for elk managers given that much of the surface area in these areas is privately owned. Limitations of sparse forest combined with private land ownership may confound efforts by natural resource agencies to manage elk herds in similar areas if not interconnected with other elk herds.

## Supporting information

S1 Fig Percent cover of each included land class of random points inside each buffer, based on ~9,000 points inside 1 km, 20,000 inside 2 km, 40,000 inside 4 km, and 80,000 inside 8 km in the Turtle Mountain study area, Bottineau and Rolette counties, North Dakota, USA (data collected from 2016–17). (TIF)

S2 Fig Percent cover of each included land class of random points inside each buffer, based on 10,000 points inside 1 km, 20,000 inside 2 km, 40,000 inside 4 km, and ~75,000 inside 8 km in the Pembina Hills study area, Cavalier and Pembina counties, North Dakota, USA (data collected from 2016–17). (TIF)

S3 Fig Percent cover of each included land class of random points inside each buffer, based on 10,000 points inside 1 km, 20,000 inside 2 km, 40,000 inside 4 km, and ~75,000 inside 8 km in the Porcupine Hills study area, Sioux County, North Dakota, USA (data collected from 2016–17). (TIF)

S4 Fig Estimated back-transformed probabilities of land class use with 95% CI for individual elk ID with random points for comparison in the Turtle Mountain study area, Bottineau and Rolette counties, North Dakota, USA (data collected from 2016–17). (TIF)

S5 Fig Estimated back-transformed probabilities of land class use with 95% CI for diel period with random points for comparison in the Turtle Mountain study area, Bottineau and Rolette counties, North Dakota, USA (data collected from 2016–17). (TIF)

S6 Fig Estimated back-transformed probabilities of land class use with 95% CI for season with random points for comparison in the Turtle Mountain study area, Bottineau and Rolette counties, North Dakota, USA (data collected from 2016–17). (TIF)

S7 Fig Estimated back-transformed probabilities of land class use with 95% CI for diel period with random points for comparison in the Pembina Hills study area, Cavalier and Pembina counties, North Dakota, USA (data collected from 2016–17). (TIF)

S8 Fig Estimated back-transformed probabilities of land class utilization with 95% CI for season with random points for comparison in the Pembina Hills study area, Cavalier and Pembina counties, North Dakota, USA (data collected from 2016–17). (TIF)

S9 Fig Estimated back-transformed probabilities of land class use with 95% CI for individual elk ID with random points for comparison in the Porcupine Hills study area, Sioux County, North Dakota, USA (data collected from 2016–17). (TIF)

S10 Fig Estimated back-transformed probabilities of land class use with 95% CI for diel period with random points for comparison in the Porcupine Hills study area, Sioux County, North Dakota, USA (data collected from 2016–17). (TIF)

S11 Fig Estimated back-transformed probabilities of land class use with 95% CI for season with random points for comparison in the Porcupine Hills study area, Sioux County, North Dakota, USA (data collected from 2016–17). (TIF)

S1 Table Multinomial logit models to test for differences in use of land classes, and for departures from proportionate use based on random sampling in Turtle Mountain, Pembina Hills, and Porcupine Hills study areas, North Dakota, USA (data collected from 2016–17). Response variable was land class associated with each location (elk observation or random point). Predictor variables were all categorical fixed effects and included elk ID (5 categories per herd), diel period (day or night), and season (summer, archery, gun, winter, calving), and any two-way interaction terms. Study area, model rank, variables, Akaike’s Information Criterion (AIC), ΔAIC, and Akaike weights (ω_i_) for top 4 logistic regression models.
(DOCX)
