# Supplementary figures and images for: Seasonal home ranges and habitat selection of three elk (Cervus elaphus) herds in North Dakota
Source: PLoS One. 2019 Feb 4;14(2):e0211650. doi: 10.1371/journal.pone.0211650 (PMC6361512; doi:10.1371/journal.pone.0211650)

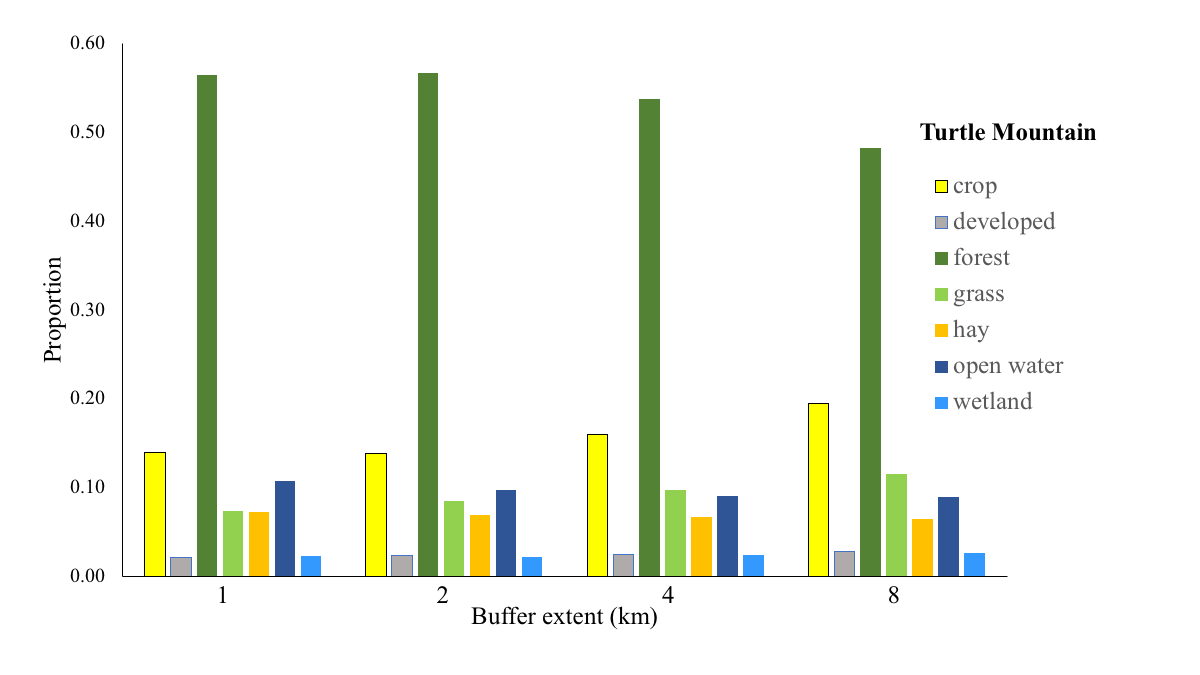

Supplement: S1 Fig — (TIF) [file pone.0211650.s001.tif]

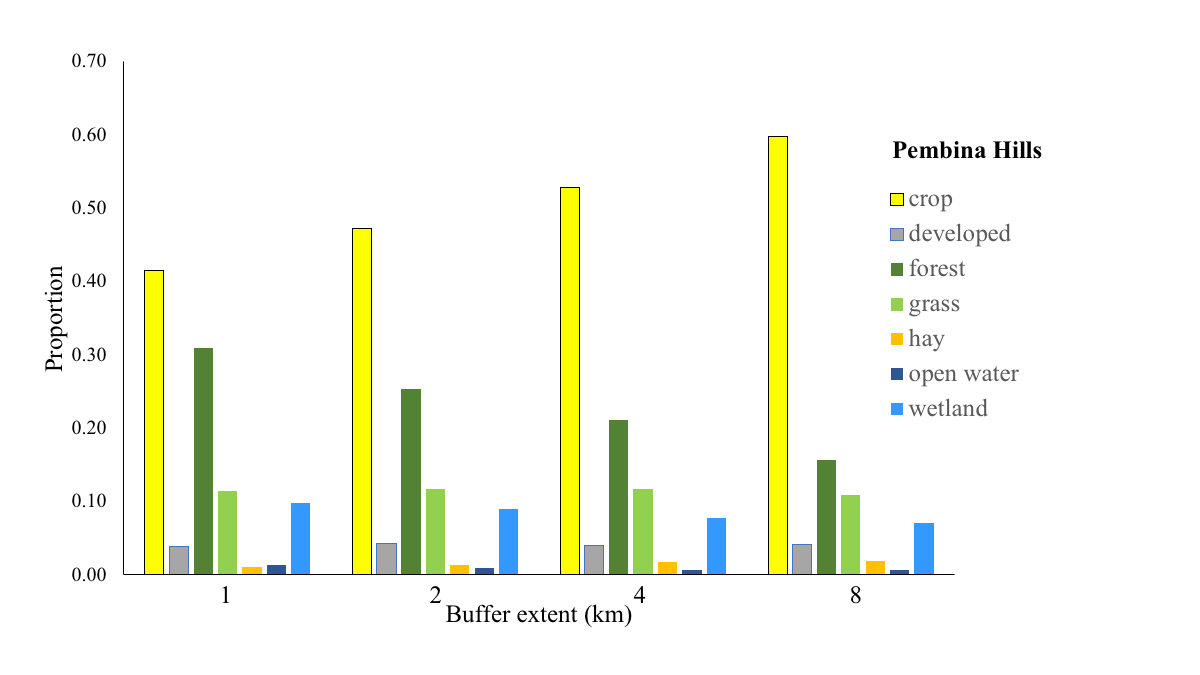

Supplement: S2 Fig — (TIF) [file pone.0211650.s002.tif]

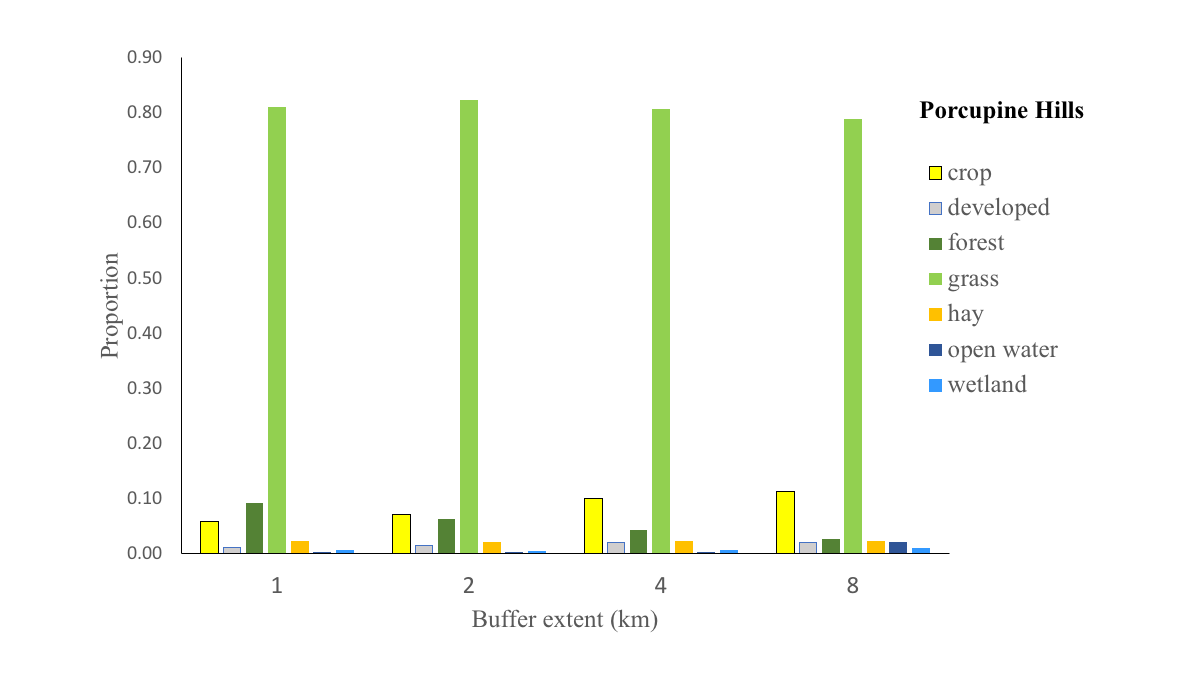

Supplement: S3 Fig — (TIF) [file pone.0211650.s003.tif]

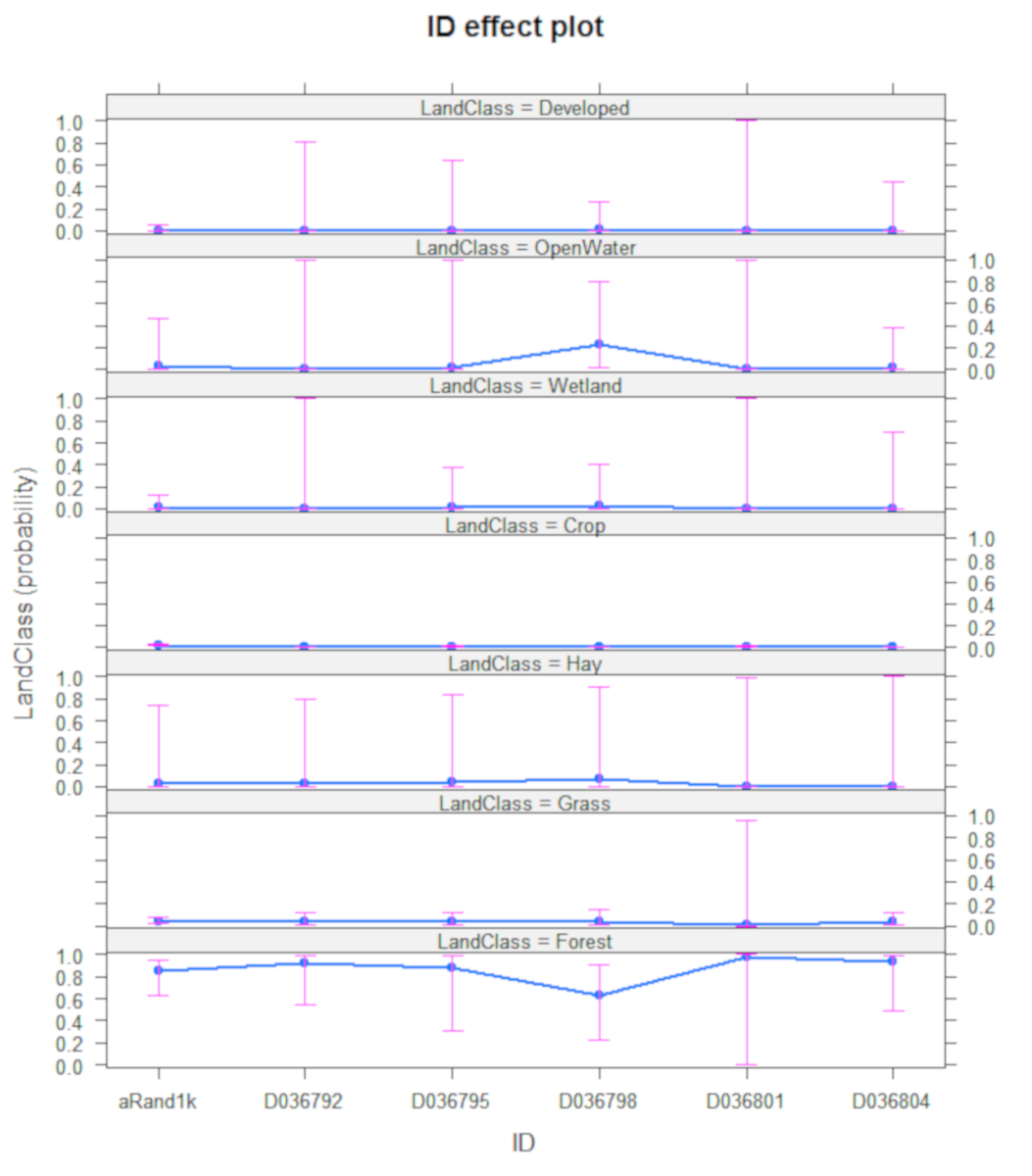

Supplement: S4 Fig — (TIF) [file pone.0211650.s004.tif]

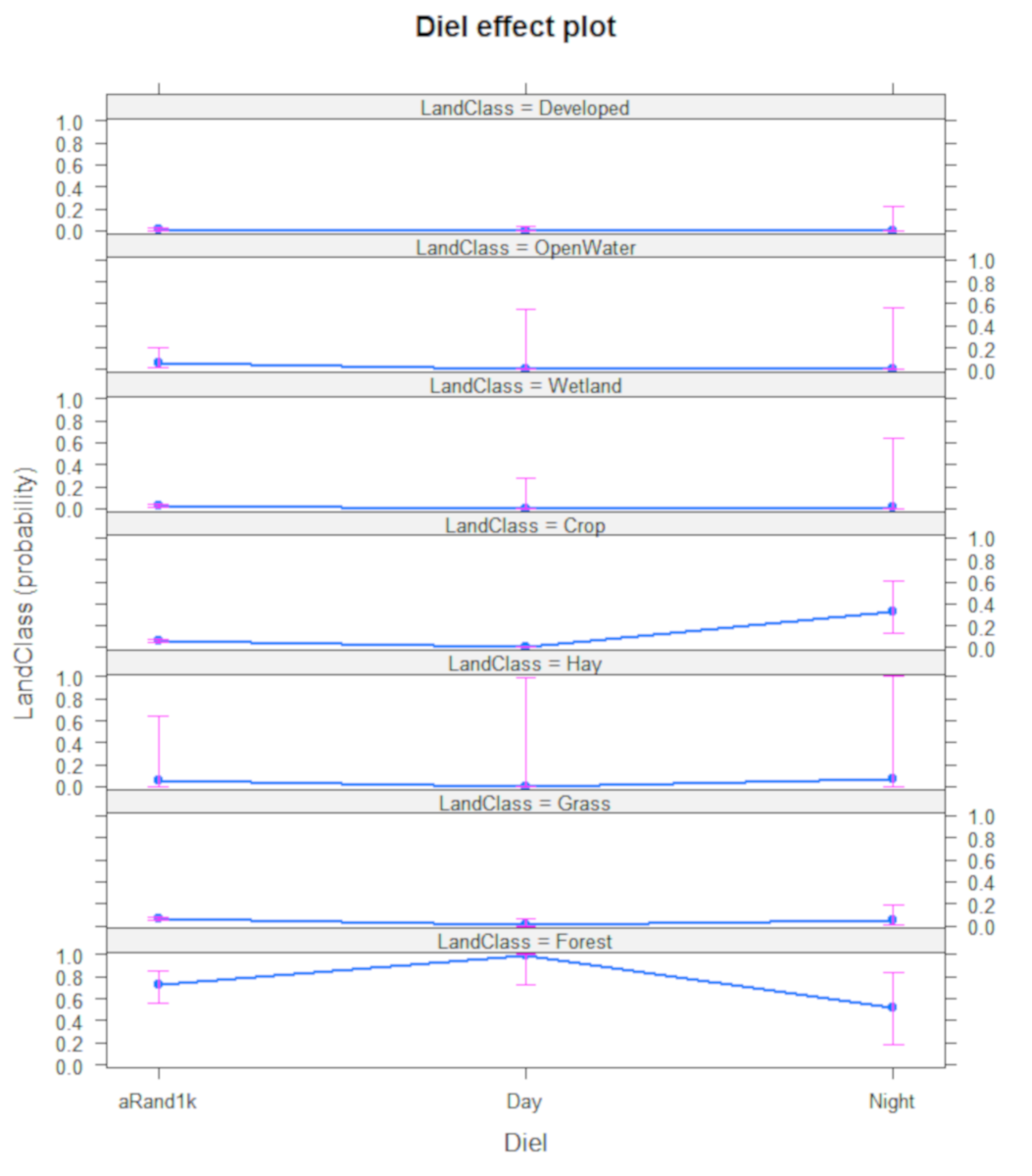

Supplement: S5 Fig — (TIF) [file pone.0211650.s005.tif]

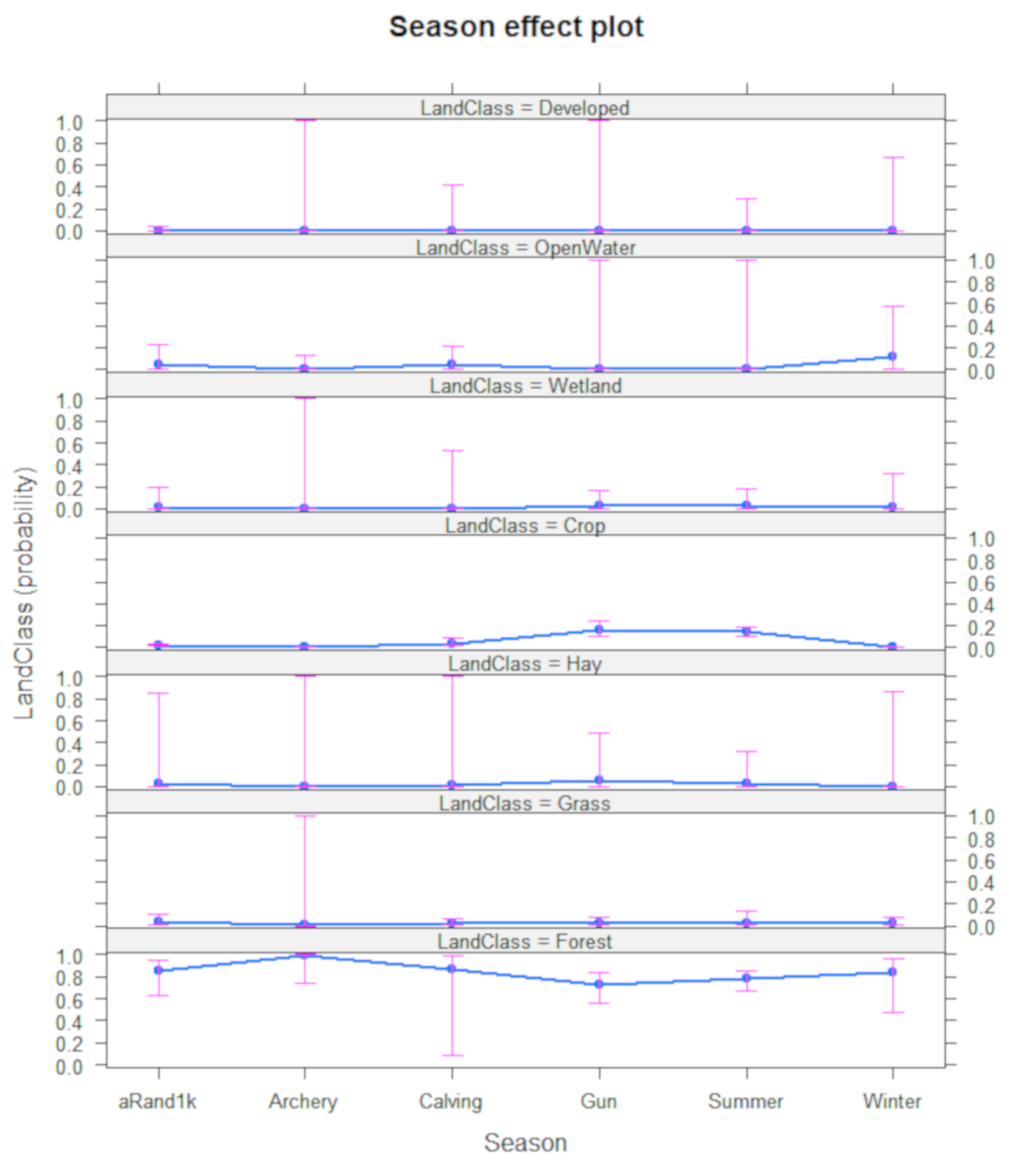

Supplement: S6 Fig — (TIF) [file pone.0211650.s006.tif]

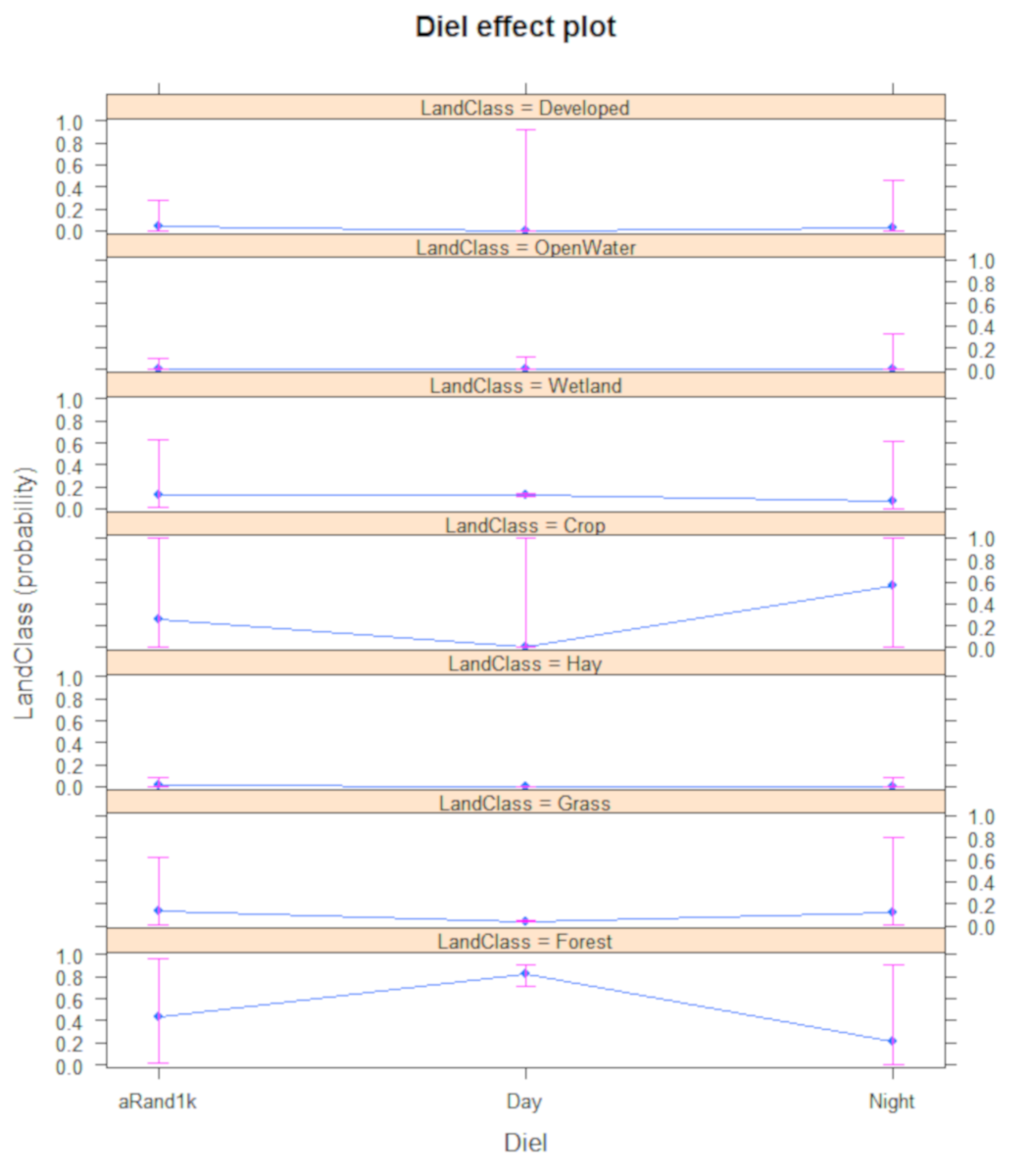

Supplement: S7 Fig — (TIF) [file pone.0211650.s007.tif]

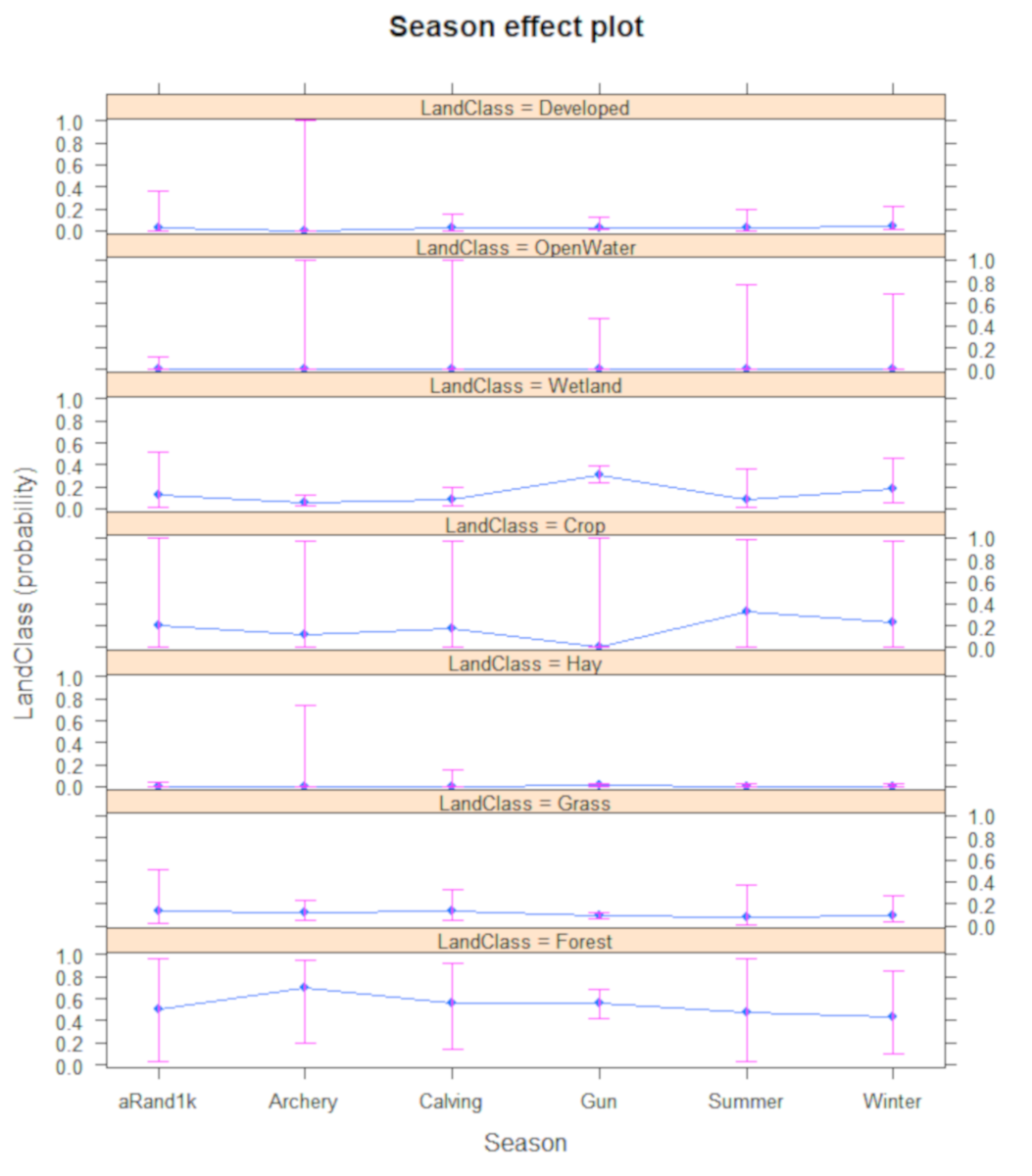

Supplement: S8 Fig — (TIF) [file pone.0211650.s008.tif]

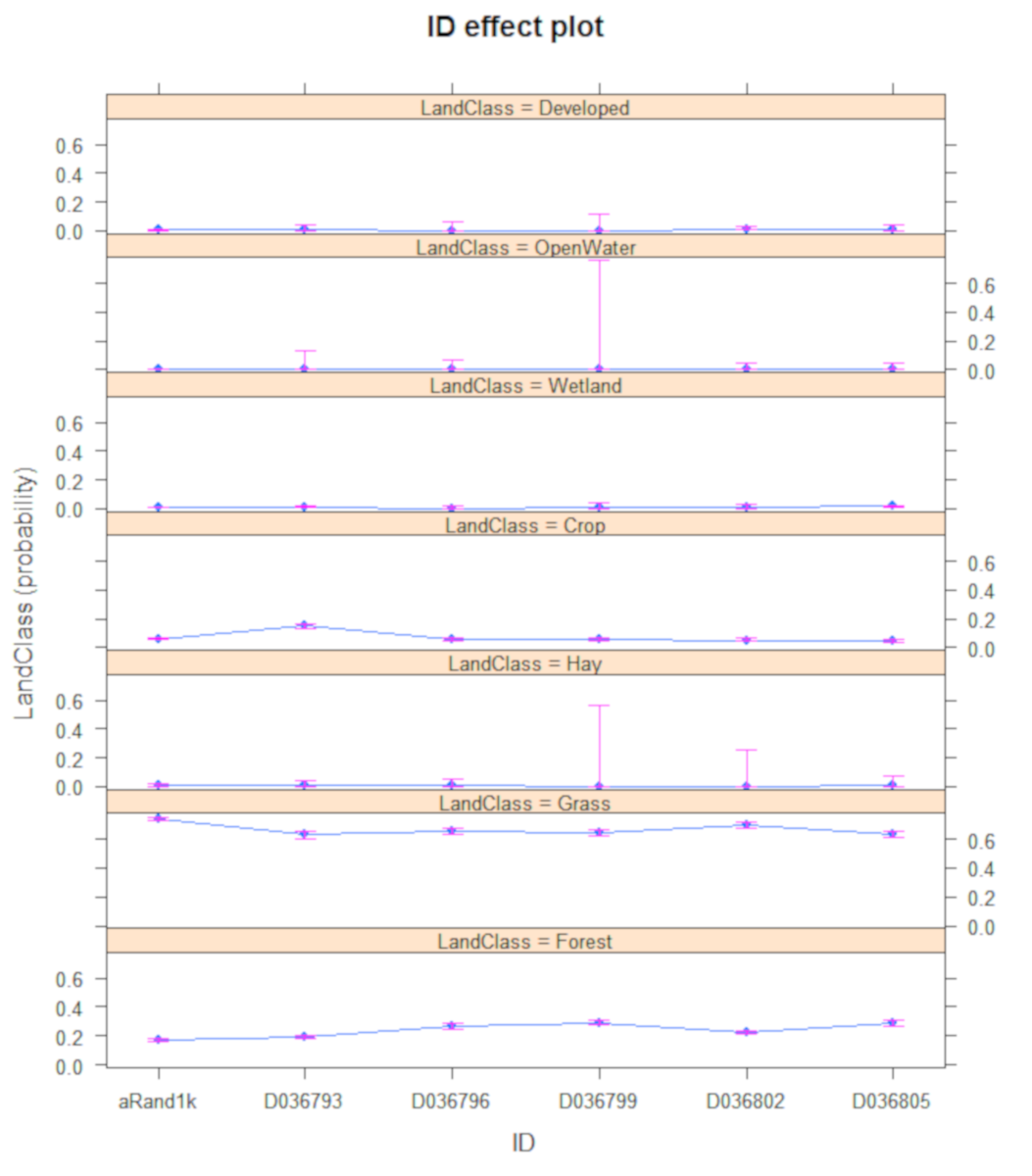

Supplement: S9 Fig — (TIF) [file pone.0211650.s009.tif]

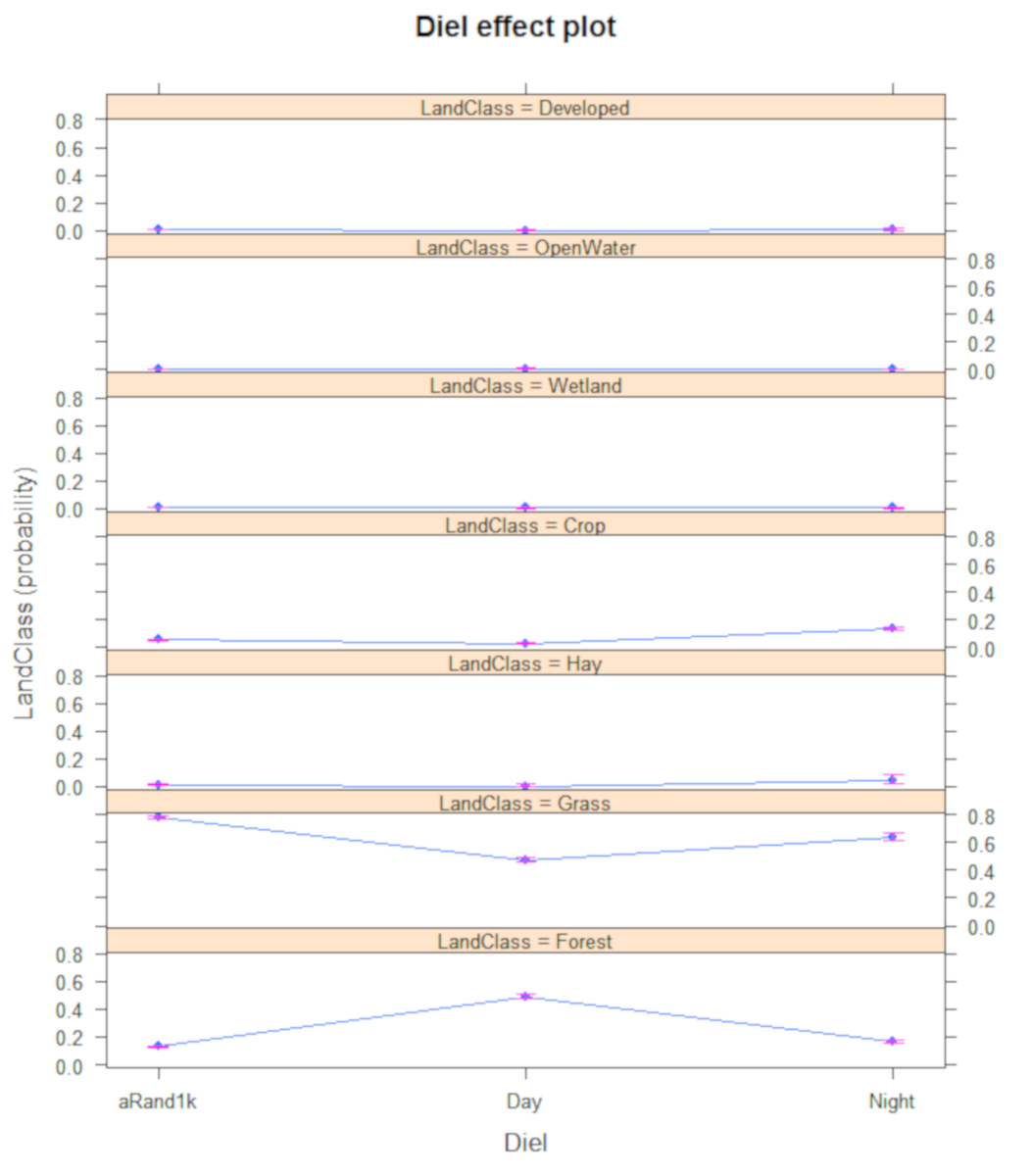

Supplement: S10 Fig — (TIF) [file pone.0211650.s010.tif]

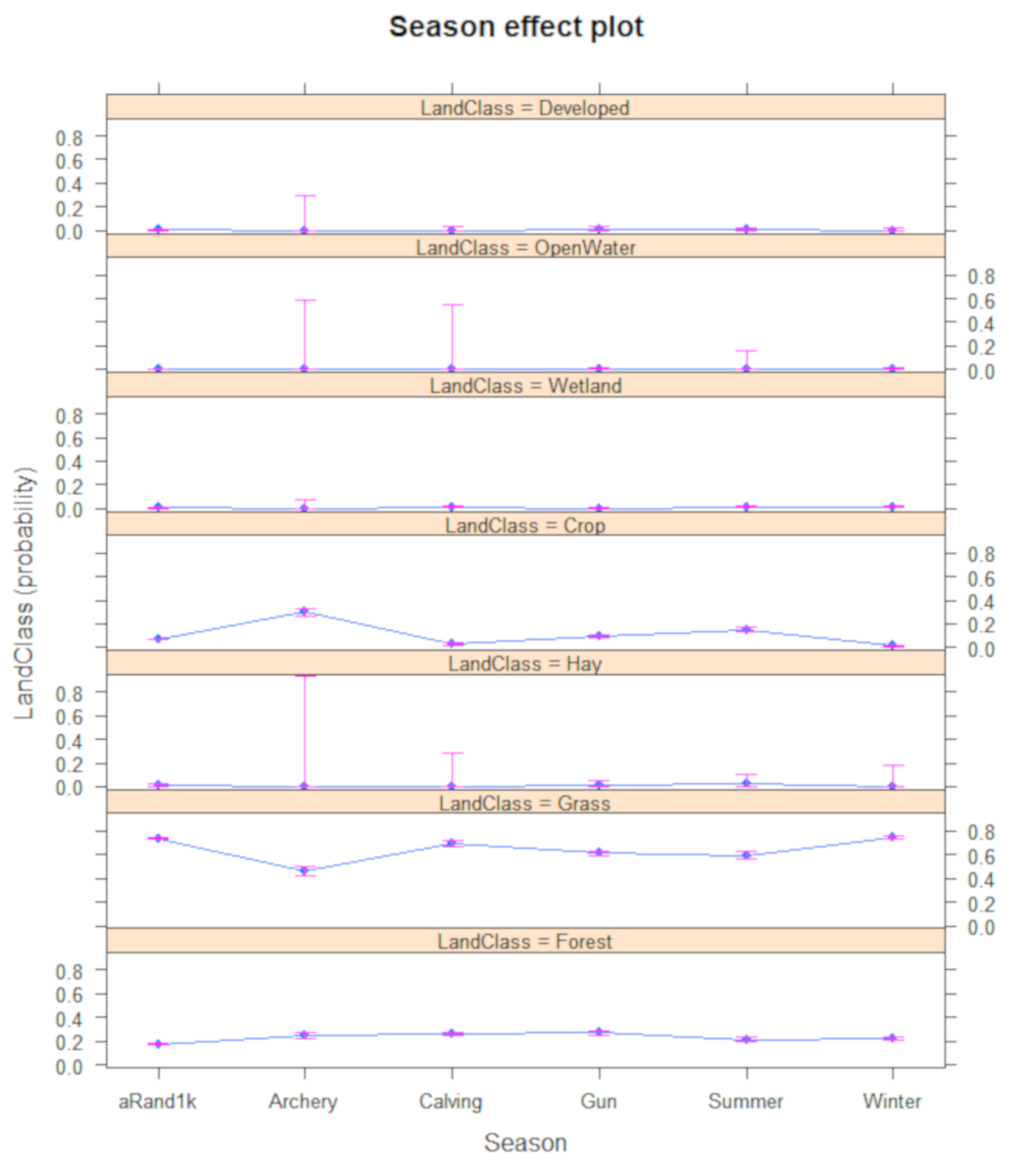

Supplement: S11 Fig — (TIF) [file pone.0211650.s011.tif]
