# Supplementary material for: Seasonal home ranges and habitat selection of three elk (Cervus elaphus) herds in North Dakota
Source: PLoS One. 2019 Feb 4;14(2):e0211650. doi: 10.1371/journal.pone.0211650 (PMC6361512; doi:10.1371/journal.pone.0211650)
Supplement: S1 Table — Response variable was land class associated with each location (elk observation or random point). Predictor variables were all categorical fixed effects and included elk ID (5 categories per herd), diel period (day or night), and season (summer, archery, gun, winter, calving), and any two-way interaction terms. Study area, model rank, variables, Akaike’s Information Criterion (AIC), ΔAIC, and Akaike weights (ωi) for top 4 logistic regression models. (DOCX) [file pone.0211650.s012.docx]

| Study area | Rank | Model Variables | AIC_c_ | ΔAIC_c_ | ω_i_ |
| --- | --- | --- | --- | --- | --- |
| Turtle Mountain | 1 | ID, Diel, Season, Diel*Season, ID*Season | 37510.0 | 0.00 | 1.000 |
|  | 2 | ID, Diel, Season, Diel*Season, ID*Season, ID*Diel | 37539.4 | 29.39 | 0.000 |
|  | 3 | ID, Diel, Season, Diel*Season | 37831.8 | 321.84 | 0.000 |
|  | 4 | ID, Diel, Season | 37976.9 | 466.89 | 0.000 |
| Pembina Hills | 1 | ID, Diel, Season, Diel*Season, ID*Season | 47774.1 | 0.00 | 1.000 |
|  | 2 | ID, Diel, Season, Diel*Season, ID*Season, ID*Diel | 47804.9 | 30.75 | 0.000 |
|  | 3 | ID, Diel, Season, Diel*Season | 47937.6 | 163.42 | 0.000 |
|  | 4 | ID, Diel, Season | 48119.1 | 344.94 | 0.000 |
| Porcupine Hills | 1 | ID, Diel, Season, Diel*Season, ID*Season, ID*Diel | 34093.0 | 0.00 | 0.991 |
|  | 2 | ID, Diel, Season, Diel*Season, ID*Season | 34102.3 | 9.31 | 0.009 |
|  | 3 | ID, Diel, Season, Diel*Season | 34250.9 | 157.85 | 0.000 |
|  | 4 | ID, Diel, Season | 34315.0 | 221.97 | 0.000 |
